# Novel MHC *BLB2* gene polymorphism and its association with IgY concentration and Newcastle disease antibody titer in IPB-D2 chickens 

**DOI:** 10.5194/aab-66-275-2023

**Published:** 2023-09-21

**Authors:** Dwi Lestari, Sri Murtini, Niken Ulupi, Asep Gunawan, Cece Sumantri

**Affiliations:** 1 Graduate School of Animal Production and Technology, Faculty of Animal Science, IPB University, Bogor, 16680, Indonesia; 2 Department of Animal Disease and Veterinary Public Health, Faculty of Veterinary Medicine, IPB University, Bogor, 16680, Indonesia; 3 Department of Animal Production and Technology, Faculty of Animal Science, IPB University, Bogor, 16680, Indonesia

## Abstract

This study aimed to identify the polymorphism of the B Locus Beta 2 (*BLB2*) gene and its association with immunoglobulin Y (IgY) concentration and Newcastle disease (ND) antibody titer; we analyzed *BLB2* gene expression in different categories of ND antibody titers in IPB-D2 chickens. The total sample used was 100 IPB-D2 chickens. Blood samples were collected at 21 weeks old for an ELISA (enzyme-linked immunoassay) test, an HI (hemagglutination inhibition) test, and genotyping. The method for *BLB2* polymorphism was Sanger sequencing. Analysis of *BLB2* gene expression was performed using the cecal tonsil tissue of IPB-D2 chickens. Polymorphism data were analyzed using SNPstats and DNAsp (DNA Sequence Polymorphism) software. The association of the single-nucleotide polymorphisms (SNPs) with IgY concentration and ND antibody titer was analyzed using SAS software (version 9.2). The genotype mean values were compared by means of a T test. The relative mRNA expression analysis was performed using a quantitative real-time polymerase chain reaction (qRT-PCR). The results showed that 13 SNPs were found in exon 2 and exon 3 in the *BLB2* gene. As many as 4 out of the 13 SNPs were associated with IgY concentration. As many as 9 out the 13 SNPs may have changed amino acids. The Δ Ct value showed that the expression of the *BLB2* gene in IPB-D2 chickens with high ND antibody titers is higher than IPB-D2 chickens with low ND antibody titers. In conclusion, the AA genotype of g.458 T > A was associated with high IgY concentrations, and the *BLB2* gene presented with a high expression in IPB-D2 chickens with high ND antibody titers.

eces@apps.ipb.ac.id]CeceSumantri

## 1 Introduction

The IPB-D1 chicken is a new breed of local Indonesian chicken resulting from the crossing of F1 males of Pelung chickens and Sentul chickens with F1 females of Kampong chickens and broiler chicken parent stock (strain – Cobb). The selection of the chickens is predicted based on the fact that Pelung chickens have a large body frame with the capacity to produce more meat. Sentul chickens have a high degree of egg production, whereas Kampong chickens are resistant to *Salmonella* sp. In order to enhance growth, crosses were made with broiler chickens, which have fast-growth traits. However, the presence of 25 % broiler blood permits a decrease in chicken disease resistance traits (Ulupi et al., 2016).

The development of the new line of IPB-D2 chickens is one of the efforts to produce local chickens with good disease resistance traits. IPB-D2 chickens are selected from IPB-D1 chickens based on several immunocompetence traits such as IgY concentration and Newcastle disease (ND) antibody titer. IPB-D2 chickens were selected based on IgY concentration ≥10 mg mL ${}^{-1}$ and Newcastle disease (ND) antibody titer $\geq 3{\mathrm{log}}_{2}$ HI unit. IgY is the main antibody of chickens in the body's response mechanism against pathogens. IgY is found in the blood and is inherited as an antibody in chicks. Meanwhile, ND antibody titer is an illustration of the protective immune response against Newcastle disease (Rahman et al., 2017).

In Indonesia, local chickens are still raised using a traditional system with extensive systems, where chickens are allowed to roam freely outdoors and forage naturally. This rearing system increases the chance of chickens being exposed to pathogens that can lead to disease and mortality. A disease occurs when the immune system fails to defend the body against the effect of invading pathogens (Zekarias et al., 2002). The immune system is also affected by genetic factors in addition to environmental factors. Genes produce certain proteins that play a role in influencing the chicken's immune system.

The major histocompatibility complex (MHC) is a region of genes that controls immune responses, and it is found in all vertebrate species (Miller and Taylor, 2016). There are class-I (BF), class-II (BL), and class-IV (BG) genes in the chicken MHC (B complex) gene cluster, which is on microchromosome 16 (Lamont, 1991). The highly polymorphic classical class-I and class-II molecules of the major histocompatibility complex (MHC) play important roles in the adaptive immune system by presenting peptides to T cells. They also play important roles in the innate immune system as ligands for natural killer (NK) cells (Kaufman, 2022). The chicken MHC is strongly linked to resistance and susceptibility to pathogens, which is important to the economy.

Based on the sequence of the polymorphic region 1, the various B-LB gene isotypes can be classified into three families: the B-LB II family (consisting of B-LB I and BL-B II), the B-LB III family (consisting of B-LB III, IV, and V), and the B-LB VI family (Zoorob et al., 1990). Similarly to the mammalian MHC class-II beta chain gene, the B-LB II gene has been widely researched because of its widespread polymorphism and crucial function in the presentation of extracellular antigen peptides to helper T cells, which is the beginning of the immune response and the thymic selection of T lymphocytes (Xu et al., 2007).

The MHC class II is divided into two groups of chains: α and β . The class-II β chains are encoded by either B Locus Beta 1 (BLB1) or B Locus Beta 2 (*BLB2*). Diversity of the MHC class-II antigen-binding region is crucial for the suppression of adaptive immunological responses (Zekarias et al., 2002). It has been found that exon 2 of *BLB1* and *BLB2*, which encodes for the antigen-binding region domain, is very variable (Li et al., 2010). Niikura et al. (2004) suppose that *BLB1* and *BLB2* play a role in disease resistance (e.g., against Marek's disease and salmonellosis) (Liu et al., 2002; Zhou and Lamont, 2003).

MHC is a polymorphic region that has numerous single-nucleotide polymorphisms (SNPs) and insertions and deletions (INDELs). Yuan et al. (2021) found 3319 SNPs and 181 INDELs in the BF and BL regions among 21 chicken populations, of which 2057 SNPs and 159 INDELs were novel. In three local Chinese breeds, most of the mutation positions were located in the B-LB β 1 domain encoded by exon 2, especially in the peptide-binding region (Chen et al., 2012). Chen et al. (2012) also reported that the chicken BL gene showed more polymorphic sites and clearly dominant trans-breed alleles, potentially to adapt to pathogens. The study of Lestari et al. (2022) stated that SNPs in the MHC class-II *DMA* gene had an association with IgY concentration in IPB-D2 chickens.

The *BLB2* gene is one of the polymorphic genes. The *BLB2* gene is located between Tapasin and RING3 in the BL region. The *BLB2* gene plays an important role in extracellular antigen presentation and in the initiation of the immune response (Guo et al., 2012). Niikura et al. (2004) stated that, based on the results of gene mapping, the *BLB2* gene is a candidate gene that affects the immune system of chickens. The *BLB2* gene is 1573 bp long and consists of seven exons and six introns. Several studies related to the diversity of the *BLB2* gene have been carried out. There were nine genotypes of the *BLB2* gene in Silkies (Qianyun et al., 2000) and 31 new alleles of the *BLB2* gene in native Chinese chickens (Xu et al., 2007). In addition, the *BLB2* gene is well expressed in all chicken tissues such as the cecal tonsils, cecum, spleen, duodenum, brain, and lungs (Parker and Kaufman, 2017).

Analysis of MHC allelic polymorphism in other breeds is uncommon, particularly in the Indonesian local chickens and especially in IPB-D2 chickens. The aim of this study was mainly to focus on the following: (1) characterizing the genetic polymorphism of the chicken MHC *BLB2* gene in IPB-D2 chickens, (2) providing information about *BLB2* gene polymorphism and its association with IgY concentration and ND antibody titer, and (3) analyzing *BLB2* gene expression in different categories of ND antibody titers in IPB-D2 chickens.

**Table 1:** *BLB2* and *GAPDH* gene primers for relative mRNA expression.

No.	Gene	Primer sequences	GenBank accession	PCR product
			number	(bp)
1	*BLB2*	F: 5 ${}^{\prime }$ -GCACAACTACGGGATTCTGG-3 ${}^{\prime }$ R: 5-TCAGGAACCACTTCACCTCG-3 ${}^{\prime }$	NM_001318995	161
2	*GAPDH*	F: 5 ${}^{\prime }$ -CACTGTCAAGGCTGAGAACG-3 ${}^{\prime }$ R: 5-GCTTAGCACCACCCTTCAGA-3 ${}^{\prime }$	NM_204305.1	179

## 2 Material and methods

### 2.1 Animals and blood collection

IPB-D2 chickens were reared in an intensive system and fed twice a day, in the morning and in the evening. The feed given was 100 % commercial feed for chickens up to 4 weeks old and commercial feed and rice bran at a ratio of 70 : 30 for chickens at 4 to 12 weeks old. Chickens at 12 to 21 weeks old were given commercial feed and bran at a ratio 60 : 40. Drinking water was given ad libitum. Chickens were kept in a cage with facilities for feeding, drinking water, laying eggs, and husks.

The total sample used was 100 IPB-D2 chickens. The blood samples were collected at 21 weeks using 3 mL syringe in the venae brachiales (the brachial veins). The experimental procedure was approved by the Institutional Animal Care and Use Committee (IACUC) at IPB University (approval ID no. 224-2021 IPB).

### 2.2 ELISA test

IgY concentration was determined by means of an indirect ELISA (enzyme-linked immunoassay) method based on that of Vansofla et al. (2021) with some modifications. We used 96-well plates that were coated with IgG goat anti-IgY (SAB3700195 Sigma-Aldrich, 2.5 µ g m L ${}^{-1})$ diluted in bicarbonate buffer (Na ${}_{2}$ NO ${}_{3})$ with a pH of 9.6 at 4 ${}^{\circ }$ C overnight. The, the plates were washed three times using phosphate-buffered saline (PBS) containing Tween 20 (PBST-20, pH 7.4) and blocked with 100 µ L of 2 % bovine serum albumin (BSA) for 1 h at 37 ${}^{\circ }$ C. Serum samples were diluted at a ratio of 1 : 100 and were added to each well and incubated for 1 h at 37 ${}^{\circ }$ C.

The plates were washed three times with PBST, and 100 µ L of secondary antibody IgG rabbit anti-IgY (A9046 Sigma-Aldrich) was added to each well conjugated with a peroxide enzymes. The plate was incubated for 1 h at 37 ${}^{\circ }$ C and then washed three times using PBST. Then 100 µ L of tetramethylbenzidine (TMB) substrate solution (T0440 Sigma-Aldrich) was added to each well. Finally, the reaction was stopped with stop solution H ${}_{2}$ SO ${}_{4}$ , and the absorbance was read at 450 nm on a microplate reader (Bio-Rad, USA).

### 2.3 HI Test

The ND antibody titer was determined by means of an HI (hemagglutination inhibition) test based on the guidelines of the Office International des Epizooties (OIE, 2021) with some modifications. The determination of the antibody titer was based on the resistance in the dilution which is able to bind antigens at a concentration of 4HAU and to inhibit red blood cell agglutination. The first step was to add 25 µ L of PBS into the microplate, followed by the addition of 25 µ L of the serum sample into the first row of microplate wells and then serial dilution to the 11th well. The ND antigen was then added into every well except the 12th well. The microplate was then incubated at room temperature for 30 min. Then, 25 µ L of 1 % RBC (red blood cell count) was added up to the 12th well. RBCs and antigen were present in the 11th as a positive control, whereas RBCs alone were present in the 12th well as a negative control. By gently shaking the microplate, the RBCs were allowed 40 min to settle at room temperature. A sharp button that appeared as a result of the settlement of intact RBCs was recorded as a positive test result after the test result was assessed by titling the plates. The maximum dilution of each sample was taken into consideration as the test's endpoint, and the serum antibody titer was calculated from that by measuring the observed result in reverse.

**Figure 1:**
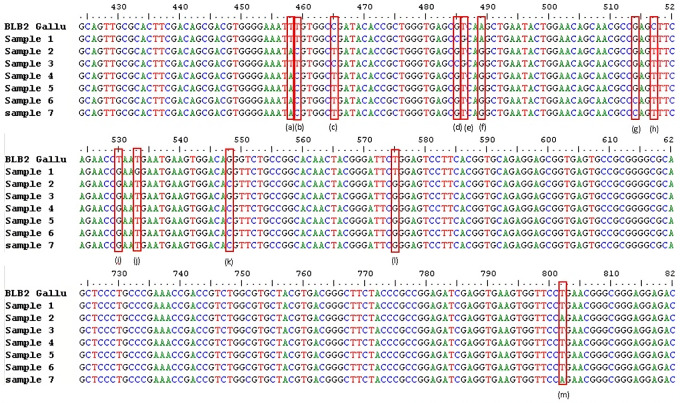
SNPs of *BLB2* gene in IPB-D2 chickens. **(a)** g.458 T > A, **(b)** g.459 T > C, **(c)** g.465 C > T, **(d)** g.485 G > C, **(e)** g.486 T > G, **(f)** g489 A > G, **(g)** g.514 G > C, **(h)** g.517 C > T, **(i)** g.530 T > G, **(j)** g.533 T > G, **(k)** g.548 G > C, **(l)** g.575 T > G, and **(m)** g.802 T > A. Exon 2: 332–601. Intron 2: 602–689. Exon 3: 690–971.

### 2.4 Sequencing analysis

DNA samples were extracted from the fresh blood using the genomic DNA extraction mini kit (Geneaid™, Taiwan) according to the manufacturer's instruction. The target of *BLB2* gene amplification is an 871 bp DNA sequence covering a part of exon 2 to exon 4 (NC_006103). The primer sequences are as follows: F – 5 ${}^{\prime }$ -GTGAGGTTTCTGGACAGG-3 ${}^{\prime }$ and R – 5 ${}^{\prime }$ -CCTGAAACACAGCGAGAC-3 ${}^{\prime }$ . The primers were designed using the PCR Primer Stats program.

The final volume of the *BLB2* gene PCR was 25 µ L with a concentration of 20 pmol µ L ${}^{-1}$ , consisting of 1 µ L DNA sample, 9.75 µ L distilled water (DW), forward and reverse primers of 0.25 µ L, a MyTaq™ HS Red Mix of 12.5 µ L, and 1.25 µ L dimethyl sulfoxide (DMSO) (5 %). DNA was amplified using a PCR machine with the following procedure: pre-denaturation at 95 ${}^{\circ }$ C for 1 min, denaturation at 95 ${}^{\circ }$ C for 15 s, annealing at 56 ${}^{\circ }$ C for 15 s, extension at 72 ${}^{\circ }$ C for 10 s, and final extension 72 ${}^{\circ }$ C for 3 min. PCR products were visualized in 1 % agarose gel. The sequencing analysis was outsourced to Macrogen, Korea.

### 2.5 Relative mRNA Expression of *BLB2* gene

The sample used for *BLB2* gene expression was the cecal tonsil of IPB-D2 chickens. Samples were used based on differences between high ND antibody titers (3.25 ± 0.25 log ${}_{2}$ HI unit) and low ND antibody titers (1.24 ± 1.15 log ${}_{2}$ HI unit). RNA extraction was performed according to the protocol of the kit used (RNeasy Mini Kit, Qiagen). RNA purity was analyzed using NanoDrop™ 2000/2000c Spectrophotometers (Thermo Fisher Scientific). The RNA used was RNA with an A260 / A280 ratio value of 1.8–2.2. Complementary DNA (cDNA) was synthesized using a First Strand cDNA Synthesis Kit (Thermo Fisher Scientific). The quantification of cDNA was performed using the qRT-PCR (quantitative real-time polymerase chain reaction) method. The qRT-PCR mix consist of 2 µ L of cDNA sample, 3 µ L of DW, 0.5 µ L of forward primer, 0.5 µ L of reverse primer, and 5 µ L of SBYR^®^ Green (Toyobo THUNDERBIRD^®^ SBYR^®^ qPCR Mix). The quantification was carried out with pre-denaturation at 95 ${}^{\circ }$ C for 1 min, denaturation at 95 ${}^{\circ }$ C for 15 s, and annealing at 57 ${}^{\circ }$ C for 1 min with 40 repetitions. The specific primers used were designed using PCR Primer Stats software (Table 1). The housekeeping gene used was glyceraldehyde 3-phosphate dehydrogenase (*GAPDH*). The relative expression of the *BLB2* gene mRNA was calculated based on the delta Ct ( Δ Ct) according to Silver et al. (2006) with the following formula:

ΔCt=Ct target gene-Ct housekeeping gene.

### 2.6 Statistical analysis

The results of DNA sequencing were analyzed using FinchTV, MEGA X, and BioEdit. Allele and genotype frequencies and the Hardy–Weinberg analysis of the *BLB2* gene were analyzed using SNPstats (Pan et al., 2019). The observed heterozygosity ( $H_{\text{o}}$ ) and expected heterozygosity ( $H_{\text{e}}$ ) were analyzed using DNAsp. The association of the SNPs with IgY concentration and ND antibody titer were analyzed using SAS software (version 9.2). The genotype mean values were compared with a T test.

**Table 2:** Amino acids changed in *BLB2* gene in IPB-D2 chickens.

No.	SNPs	Amino acid changed
1	g.458 T > A	Phenylalanine > phenylalanine
2	g.459 T > C	
3	g.465 C > T	Alanine > alanine
4	g.485 G > C	Arginine > proline
5	g.486 T > G	
6	g.489 A > G	Glutamine > glutamine
7	g.514 G > C	Glutamic acid > glutamine
8	g.517 C > T	Leucine > phenylalanine
9	g.530 T > G	Leucine > arginine
10	g.533 T > G	Methionine > arginine
11	g.548 G > C	Arginine > threonine
12	g.575 T > G	Leucine > arginine
13	g.802 T > A	Leucine > glutamine

## 3 Results

The analysis of the *BLB2* gene polymorphism in IPB-D2 chickens was analyzed using the Sanger sequencing method. Figure 1 shows the *BLB2* gene polymorphism in IPB-D2 chickens. There were 13 SNPs in the *BLB2* gene, with 12 SNPs in exon 2 and 1 SNP in exon 3. The SNPs in the *BLB2* gene are thought to change some of the amino acids formed (Table 2). There are four SNPs that are synonymous, namely, g.458 T > A, g.459 T > C, g.465 C > T, and g.489 A > G.

**Table 3:** Allele frequency, genotype frequency, heterozygosity, and Hardy–Weinberg equilibrium of *BLB2* gene polymorphism in IPB-D2 chickens.

SNPs	Allele frequency		Genotype frequency			$H_{\text{o}}$	$H_{\text{e}}$	$x^{2}$
	A	B	AA	AB	BB			
g.458 T > A	0.05	0.95	0.05		0.95	0	0.0873	<0.0001
g.459 T > C	0.05	0.95	0.05		0.95	0	0.0873	<0.0001
g.465 C > T	0.02	0.98	0.02		0.98	0	0.0447	<0.0001
g.485 G > C	0.97	0.03	0.97		0.03	0	0.0662	<0.0001
g.486 T > G	0.97	0.03	0.97		0.03	0	0.0662	<0.0001
g.489 A > G	0.02	0.98	0.02		0.98	0	0.0447	<0.0001
g.514 G > C	0.98	0.02	0.95		0.05	0.0455	0.0447	<0.0001
g.517 C > T	0.03	0.97	0.03		0.97	0	0.0662	1
g.530 T > G	0.03	0.97	0.02	0.01	0.97	0.0114	0.0555	<0.0001
g.533 T > G	0.98	0.02	0.95		0.05	0.0455	0.0447	1
g.548 G > C	0.03	0.97	0.03		0.97	0	0.0664	<0.0001
g.575 T > G	0.02	0.98	0.02		0.98	0	0.0447	<0.0001
g.802 T > A	0.55	0.45	0.41	0.27	0.32	0.2727	0.4987	<0.0001

The allele and genotype annotations use the letters A and B, with A being the allele according to the reference and B being the allele mutation. AB is heterozygous mutation, and BB is homozygous mutation.

**Table 4:** Association of *BLB2* gene with IgY concentration and ND antibody titer in IPB-D2 chickens.

SNPs	Geno-	Parameters	
	type		
		IgY concentration	ND antibody titer
		(mg mL ${}^{-1})$ ( n )	(log ${}_{2}$ HI unit) ( n )
g.458 T > A	TT	10.62 ± 0.75 (4) ${}^{\text{a}}$	1 ± 0.58 (3)
	TA		
	AA	12.69 ± 0.21 (84) ${}^{\text{b}}$	1.57 ± 0.17 (77)
g.459 T > C	TT	11.23 ± 1.08 (4)	1.5 ± 0.65 (4)
	TC		
	CC	12.66 ± 0.21 (84)	1.55 ± 0.17 (76)
g.465 C > T	CC	10.89 ± 1.54 (2)	0.5 ± 0.5 (2)
	CT		
	TT	12.63 ± 0.21 (2)	1.58 ± 0.16 (78)
g.485 G > C	GG	12.67 ± 0.21 (85) ${}^{\text{a}}$	1.57 ± 0.17 (77)
	GC		
	CC	10.42 ± 1.01 (3) ${}^{\text{b}}$	1 ± 0.58 (3)
g.486 T > G	TT	12.67 ± 0.21 (85) ${}^{\text{a}}$	1.57 ± 0.17 (77)
	TG		
	GG	10.42 ± 1.01 (3) ${}^{\text{b}}$	1 ± 0.58 (3)
g.489 A > G	AA	10.95 ± 1.49 (2)	1.5 ± 0.5 (2)
	AG		
	GG	12.63 ± 0.21 (86)	1.55 ± 0.16 (78)
g.514 G > C	GG	12.56 ± 0.22 (84)	1.53 ± 0.16 (78)
	GC	13.44 ± 0.45 (4)	2.5 ± 0.5 (2)
	CC		
g.517 C > T	CC	12.5 ± 1.52 (3)	2 (1)
	CT		
	CC	12.6 ± 0.21 (85)	1.54 ± 0.16 (79)
g.530 T > G	TT	10.95 ± 1.49 (2)	1.5 ± 0.5 (2)
	TG	11.73 (1)	4 (1)
	GG	12.64 ± 0.21 (85)	1.52 ± 0.16 (77)
g.533 T > G	TT	12.66 ± 0.2 (84)	1.51 ± 0.16 (77)
	TG	11.14 ± 1.77 (4)	2.67 ± 0.88 (3)
	GG		
g.548 G > C	GG	10.42 ± 1.01 (3) ${}^{\text{a}}$	1 ± 0.58 (3)
	GC		
	CC	12.67 ± 0.21 (85) ${}^{\text{b}}$	1.67 ± 0.17 (77)
g.575 T > G	TT	10.95 ± 1.49 (2)	1.5 ± 0.5 (2)
	TG		
	GG	12.63 ± 0.21 (86)	1.55 ± 0.16 (78)
g.802 T > A	TT	12.46 ± 0.3 (36)	1.42 ± 0.24 (33)
	TA	12.58 ± 0.53 (24)	1.77 ± 0.31 (22)
	AA	12.79 ± 0.29 (28)	1.52 ± 0.31 (25)

Means in the same column with different superscripts differ significantly between genotypes ( p<0.05 ); n is number of samples.

Table 3 shows the values of allele frequency, genotype frequency, heterozygosity, and Hardy–Weinberg equilibrium in the *BLB2* gene. All SNPs in the *BLB2* gene have three alleles that form 3 genotypes at 2 SNPs and 2 genotypes at 11 SNPs. Based on observed heterozygosity ( $H_{\text{o}}$ ) and expected heterozygosity ( $H_{\text{e}}$ ) values, SNPs in the *BLB2* gene have a smaller $H_{\text{o}}$ value than the $H_{\text{e}}$ value. The Hardy–Weinberg equilibrium is seen from the $x^{2}$ value. The $x^{2}$ values indicates that IPB-D2 chickens are not expected in the Hardy–Weinberg equilibrium.

Table 4 shows the association of the *BLB2* gene SNPs with IgY concentration and ND antibody titer. Based on statistical analysis, four SNPs were significantly associated ( P<0.05 ) with IgY concentration in IPB-D2 chickens. However, SNPs in the *BLB2* gene were not significantly associated ( P>0.05 ) with the ND antibody titer.

**Figure 2:**
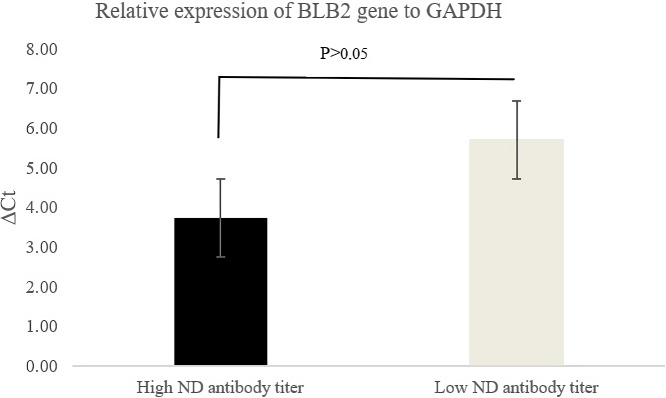
Relative expression of *BLB2* gene in IPB-D2 chickens.

Figure 2 shows the relative expression of the *BLB2* gene as indicated by the Δ Ct values. The Δ Ct value in IPB-D2 chickens with high ND antibody titers was lower than in IPB-D2 chickens with low ND antibody titers. A low Δ Ct value indicates higher expression.

## 4 Discussion

SNPs can occur in coding or non-coding regions; those that alter the amino acid sequence are known as non-synonymous SNPs, while those that do not alter the amino acid sequence are known as synonymous SNPs (Ritu and Mohapatra, 2018). *BLB2* SNPs were analyzed using the Sanger sequencing technique. The success of the *BLB2* gene sequencing was 92.6 %. A total of 13 SNPs were found in two locations: 12 SNPs in exon 2 and 1 SNP in exon 3 (Fig. 1). As many as 9 out of 13 SNPs may have caused amino acid changes in the *BLB2* gene in IPB-D2 chickens (Table 2). Research by Xu et al. (2007) found 68 SNPs in exon 2 of the *BLB2* gene in native Chinese chickens, while Guo et al. (2012) found 69 SNPs in exon 2 of the *BLB2* gene in Hebei chickens.

Base changes that occur resulted in changes in amino acids, except for the SNPs g.458 T > A, g.459 T > C, g.465 C > T, and g.489 A > G. Changes in non-synonymous bases in the coding region can cause amino acid changes. Amino acid changes can drastically change the phenotype of an individual (Ng and Henikoff, 2006). The amino acid proline contributes to immune-cell-mediated wound healing and injury recovery, partly to protect lymphocytes from apoptosis, promoting cell proliferation and increasing antibody production (Abumrad and Barbul, 2004). The immune system uses glutamine to regulate T cell proliferation, protein synthesis, the creation of cytons and antibodies, the activation of macrophages, and the inhibition of apoptosis. The amino acid arginine serves as a modulator of autoimmune disease, a signaling molecule to eliminate infections, and a regulator of cytokine production (Li et al., 2007).

Genetic diversity is defined as the genomic differences between individuals within or between populations that make each or group of organisms different from others (Ritu and Mohapatra, 2018). The genetic diversity of a population can be seen from the allele frequency, genotype frequency, and heterozygosity level. According to Allendorf et al. (2012), a population is said to be polymorphic if there are two or more alleles with an allele frequency of more than 1 %. The SNPs in the *BLB2* gene have two genotypes, except for the SNPs g.802 T > A, which has three genotypes (namely, TT, TA, and AA), and g.530 T > G, which has three genotypes (TT, TG, and GG). The highest allele frequency of the SNP g.458 T > A is allele A. For the SNP g.459 T > C, it is allele C. For the SNPs g.465 C > T, g.486 T > G, g.533 T > G, and g. 802 T > A, it is the T allele, while in other SNPs, the highest allele is the G allele. Thakur et al. (2017) found eight different genotypes in the exon 2 MHC B-LB2 family gene in Kadaknath chickens based on polymerase chain reaction sequence-specific amplification (PCR-SSP).

In addition to allele frequency and genotype frequency, genetic diversity can also be measured based on the value of heterozygosity. Tambasco et al. (2003) propose that the difference between the observed heterozygosity value ( $H_{\text{o}}$ ) and the expected heterozygosity value ( $H_{\text{e}}$ ) can be utilized as a sign that the observed population lacks genotype balance. The SNP in the *BLB2* gene has a lower $H_{\text{o}}$ value compared to the $H_{\text{e}}$ value, which is in accordance with the findings of Zhao et al. (2019); this indicates that inbreeding has occurred in a population. According to the value of $x^{2}$ , which shows that the SNP is not within the Hardy–Weinberg equilibrium, this is consistent.

There are four SNPs that have significant associations with IgY concentration, i.e g.458 T > A, g.485 G > C, g.486 T > G, and g.548 G > C. The AA genotype in SNP g.458 T > A is significantly different ( p<0.05 ) from the TT genotype and has the highest mean IgY concentration among the SNPs and other genotypes. Meanwhile, SNPs of the *BLB2* gene did not show a significant association with ND antibody titer in IPB-D2 chickens. Based on the association of SNPs in the *BLB2* gene, there are four SNPs that have associations with total IgY concentrations, namely, g.458 T > A, g.485 G > C, g.486 T > G, and g.548 G > C. Each SNP had two different genotypes: SNP g.458 T > A had the genotype TT, which was significantly different ( P<0.05 ) to the genotype AA; SNP g.485 G > C had the GG genotype GG, which was significantly different ( P<0.05 ) to the genotype CC; SNP g.486 T > G had the genotype TT, which was significantly different ( P<0.05 ) to the genotype GG; and SNP g.548 G > C had the genotype GG, which was significantly different ( P<0.05 ) to CC genotype, SNP g.458 T > A genotype AA had the highest average IgY concentration of 12.69 ± 0.21 mg mL ${}^{-1}$ . Based on statistical tests, the SNP of the *BLB2* gene was not significantly different from the ND antibody titer.

SNPs that are significantly associated with IgY concentration can be utilized as potential SNPs that mark high IgY concentration. These potential SNPs can be utilized for the selection process of IPB-D2 chickens or other chickens. After it was found out that antibodies are genetically inherited, genetic selection of antibody traits has been considered to be very beneficial. Chickens that have been genetically selected for better antibody traits can produce IgY better than the chickens that are not genetically selected. Chickens will produce IgY to provide their offspring with an effective humoral response to the most widely spread pathogens before their own immune system matures (Da Silva and Tambourgi, 2010).

The qRT-PCR analysis of the *BLB2* gene was conducted using the cecal tonsil tissue of IPB-D2 chickens with different high and low ND antibody titers. Cecal tonsils are one of the largest lymphoid organs in the gut-associated lymphoid tissue (GALT) group. The cecal tonsil is the liaison between the cecum and rectum. The GALT group consists of lymphoid nodules that form lymphoid organs (Hewajuli and Dharmayanti, 2015).

The *BLB2* gene expression was calculated using the formula Δ Ct. According to Goni et al. (2009), high Δ Ct indicates low expression, while highly expressed genes have low Δ Ct. Based on qRT-PCR analysis, the *BLB2* gene in IPB-D2 chickens with high ND antibody titers had a lower Δ Ct compared to in IPB-D2 chickens with low ND antibody titers, though it was not significantly different ( P>0.05 ) (Fig. 2).

High ND antibody titer indicates the possibility of ND virus infection in the body. The results of the *BLB2* gene expression in the cecal tonsil of IPB-D2 chickens are in line with the results of Sarson et al. (2006), who stated that the Ct of the gene in MHC class II was of a lower amount in infected chickens compared to in uninfected chickens. However, this in contrast to the findings of Lian et al. (2010), who stated that the mRNA expression level of the *BLB2* gene in the spleens of chickens infected with Marek's virus was lower than that in the spleens of chickens not infected with Marek's virus. Wosen et al. (2018) stated that MHC class-II molecules are widely expressed in lung and intestinal tissues.

## 5 Conclusions

A total of 13 SNPs of the *BLB2* gene were found in exon 2 and exon 3 of IPB-D2 chickens. All SNPs are polymorphic. Four of these SNPs were found to be associated with IgY concentrations, with the AA genotype of g.458 T > A having the highest IgY concentration. The *BLB2* gene has a high expression in IPB-D2 chickens with high ND antibody titers. SNPs that are significantly associated with IgY concentration can be utilized as potential SNPs that mark high IgY concentration. These potential SNPs can be utilized for the selection process of IPB-D2 chickens or other chickens.

## Data Availability

The data sets utilized in this article are available on request from the author.
